# Cyto- and myeloarchitectural brain atlas of the pale spear-nosed bat (*Phyllostomus discolor*) in CT Aided Stereotaxic Coordinates

**DOI:** 10.1007/s00429-020-02138-y

**Published:** 2020-09-16

**Authors:** Susanne Radtke-Schuller, Thomas Fenzl, Herbert Peremans, Gerd Schuller, Uwe Firzlaff

**Affiliations:** 1 Lehrstuhl für Zoologie, Technical University Munich, Freising, Germany; 2 Department of Psychiatry, University of North Carolina At Chapel Hill, Chapel Hill, NC 27599 USA; 3 Division of Neurobiology, Department Biology II, Ludwig-Maximilians-University Munich, Planegg-Martinsried, Germany; 4 Klinikum für Anästhesiologie und Intensivmedizin am Klinikum Rechts der Isar, TU München, Munich, Germany; 5 Department of Engineering Management, University of Antwerp, Antwerp, Belgium

**Keywords:** Chiroptera, Phyllostomatidae, Cytoarchitecture, Myeloarchitecture, AChE, NADPH

## Abstract

The pale spear-nosed bat *Phyllostomus discolor*, a microchiropteran bat, is well established as an animal model for research on the auditory system, echolocation and social communication of species-specific vocalizations. We have created a brain atlas of *Phyllostomus discolor* that provides high-quality histological material for identification of brain structures in reliable stereotaxic coordinates to strengthen neurobiological studies of this key species. The new atlas combines high-resolution images of frontal sections alternately stained for cell bodies (Nissl) and myelinated fibers (Gallyas) at 49 rostrocaudal levels, at intervals of 350 µm. To facilitate comparisons with other species, brain structures were named according to the widely accepted Paxinos nomenclature and previous neuroanatomical studies of other bat species. Outlines of auditory cortical fields, as defined in earlier studies, were mapped onto atlas sections and onto the brain surface, together with the architectonic subdivisions of the neocortex. X-ray computerized tomography (CT) of the bat’s head was used to establish the relationship between coordinates of brain structures and the skull. We used profile lines and the occipital crest as skull landmarks to line up skull and brain in standard atlas coordinates. An easily reproducible protocol allows sectioning of experimental brains in the standard frontal plane of the atlas. An electronic version of the atlas plates and supplementary material is available from 10.12751/g-node.8bbcxy

## Introduction

**Fig. 1:**
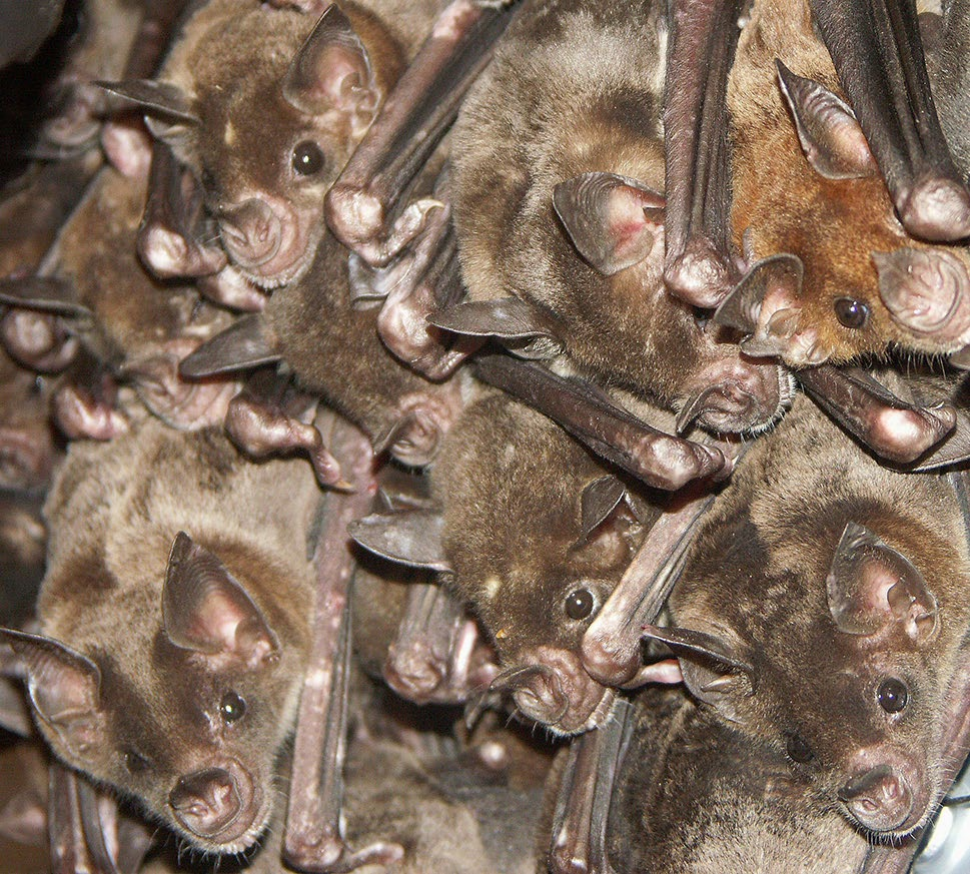
*Phyllostomus discolor* in the roost

The pale spear-nosed bat *Phyllostomus discolor* (Wagner 1843) is a medium sized microchiropteran bat with a geographic distribution ranging from Central America to the northern part of South America (Kwiecinski 2006). Body size of adult animals is ~ 10 cm with a wing span of ~ 42 cm. Based on volumetric comparisons of brain structures in bats, *Phyllostomus discolor* with its relatively large neocortex and high encephalization index has been classified as belonging to the group of ‘progressive’ chiroptera as opposed to ‘basal chiroptera’ (Pirlot and Stephan 1970; Stephan and Pirlot 1970). *Phyllostomus discolor* uses echolocation for orientation and hunting, but also olfaction and vision when lighting conditions are appropriate. *Phyllostomus discolor* is omnivorous, but mostly feeds on fruit, nectar and pollen and occasionally on insects. Echolocation calls are short (~ 1–3 ms), downward frequency modulated (90–40 kHz), multiharmonic and are emitted through the nostrils. *Phyllostomus discolor* roosts predominantly in hollow trees in colonies up to 400 individuals of both sexes (Kwiecinski 2006) (Fig. 1).

*Phyllostomus discolor* is well established as an animal model for behavioral and neurobiological research on echolocation and social communication. The peripheral and central auditory system, the vocal motor system and the visual system of *Phyllostomus discolor* have been the subject of intense research over the last decades (peripheral and central auditory system: Bartenstein et al. 2014; Esser and Kiefer 1996; Firzlaff et al. 2007; Firzlaff and Schuller 2003; Goerlitz et al. 2008; Greiter and Firzlaff 2017a, b; Heinrich et al. 2011; Hoffmann et al. 2008a, b; Linnenschmidt and Wiegrebe 2019; Vanderelst et al. 2010; vocal motor system: Fenzl and Schuller 2002; Fenzl and Schuller 2005; visual system: Hoffmann et al. 2016, 2019; Kugler et al. 2019; Rother and Schmidt 1982). However, as no brain atlas for *Phyllostomus discolor* has previously been published, only one of the earlier studies mentioned above involved neuroanatomy beyond basic localization of recording sites. The single exception was a study of the auditory cortex (AC) in *Phyllostomus discolor* (Hoffmann et al. 2008b) that identified several AC subfields based on both neurophysiological and neuroanatomical criteria.

A brain atlas is also essential for comparative studies. *Phyllostomus discolor* has become a valuable animal model in studies of the evolution of species-specific communication (Esser 1994; Esser and Schmidt 1989; Lattenkamp et al. 2018). *Phyllostomus discolor* is one of the bat species recently discovered to be capable of vocal learning (Knörnschild 2014; Vernes and Wilkinson 2020), a trait observed in only few mammalian species (Janik and Slater 1997; Tyack 2020). New approaches, including the use of molecular genetic techniques, aim to identify the neuronal substrates and brain structures underlying this behavior, for example by mapping the distribution of language related genes *FoxP1*, *FoxP2* and *CntnaP2* (Rodenas-Cuadrado et al. 2015, 2018).

The stereotaxic brain atlas of this species will enable scientists from multiple disciplines to reliably target brain structures in neurophysiological and connectional studies, and to align and compare their data with the results of immunohistochemical and molecular genetic studies utilizing a standardized anatomical representation of the *Phyllostomus discolor* brain.

Bats have a highly diverse biology and anatomy which is also observed in their brain structures, and the macromorphological differences are such, that it is nearly impossible to describe a ‘typical bat brain’ (Schneider 1957). This underlines the necessity for a distinct brain atlas of *Phyllostomus discolor*.

However, although there is considerable variety in chiropteran brains, in the preparation of our stereotaxic brain atlas for *Phyllostomus discolor,* we referred to previous brain atlases of several other bat species that were used for comparison. A cytoarchitectural atlas of the common vampire bat, *Desmodus rotundus murinus,* a species closely related to *Phyllostomus discolor,* presents frontal sections with a combined nerve fiber and cell stain (lugol fast blue-cresyl violet) and many delineated brain structures. However, although somewhat useful, there are some limitations of this earlier atlas, including poor representations of brain areas in the histological material and the absence of stereotaxic coordinates (Bhatnagar 2008). An atlas of the short-tailed fruit bat, *Carollia perspicillata* (Scalia et al. 2013), a species also closely related to *Phyllostomus discolor,* uses NeuN and Nissl as cell stains for quality sections and depicts comprehensible structural delineations of anatomical structures. High resolution frames for thalamus and amygdala are also added to this atlas. However, unfortunately, this atlas is restricted to the forebrain and also lacks a stereotaxic reference frame. A third atlas we consulted is the three-dimensional digital brain atlas of the mustached bat, *Pteronotus p. parnellii* (Washington et al. 2018) which is an MRI atlas with labels for gross brain structures. Valuable information was also gathered from the atlas of the microchiroptera brain of *Myotis montivagus*, a ‘basal chiroptera’, and the megachiropteran *Rousettus amplexicaudatus* from Baron et al. (1996) and *Rousettus aegyptiacus* (Schneider 1966).

The stereotaxic brain atlas of the bat *Phyllostomus discolor* presented here combines high-quality histological material for identification and delineation of brain structures with X-ray computerized tomography (CT). The latter yields internal contours and outlines of the skull which helped to calibrate the relationship between brain and skull coordinates. The brain atlas provides a common reference frame in stereotaxic coordinates for data from different experiments and laboratories, making it possible to reliably target brain structures for a wide range of experimental approaches. A clearly written and easily reproducible protocol provides instructions and procedures for sectioning experimental brains in the frontal plane of the atlas.

## Methods and results

### Animals

Five adult spear-nosed bats (*Phyllostomus discolor,* body weight: 40–49 g) were used for this study. The animals originated from a breeding colony in the Department of Biology II of the Ludwig-Maximilians-University in Munich. The brain of one animal was processed for cyto- and myeloarchitectural features for the atlas plates. The brains of three more animals were processed for the detection of the calcium-binding proteins parvalbumin, calbindin and calretinin, and acetylcholine-esterase, acetylcholine-transferase, NADPH-diaphorase and zinc to gather additional information for the labeling of brain structures. Two additional brain series from neurophysiological experiments stained for cytochrome oxidase were available for comparison. A CT scan of the head of one animal was performed to establish the relationship between the stereotaxic coordinates of brain structures and skull coordinates.

All experiments were conducted in accord with the NIH “Guide for the Care and Use of Laboratory Animals” (2011) and also performed in agreement with the principles of laboratory animal care and the regulations of the “German Law on Animal Protection” (209.1/211-2531-68/03 Reg. Oberbayern).

### CT imaging

The head of the animal was fixed and preserved in 4% paraformaldehyde (PFA) and then dried for approximately 20 min before scanning with a Skyscan 1076 microCT machine (Bruker, Kontich, Belgium) at the MCT group of the University Antwerpen (Belgium). Scans were performed with a resolution of 35 μm. The resulting shadow-images were processed with conebeam reconstruction software that accompanies the scanner, using a Feldkamp Reconstruction algorithm.

### Histology

Three animals were perfusion-fixed with 4% PFA. For the detection of zinc, another animal was perfused with a solution of the modified Timm method according to Danscher (1981). All animals were deeply anaesthetized with Barbital (16 mg/mL solution, 0.1 mL/10 g body weight). When a deep anesthetic state was reached, marked by a complete loss of the flexor reflex at lower limbs and wings, the animals were perfused transcardially with 0.9% saline (supplemented with 0.1% heparin) followed by 4% PFA (in 0.05 M PBS, pH 7.4).

The angle between the head and body axis during perfusion was carefully positioned in the atlas animal, to be about 110° since this angle influences the macroscopic orientation of the most caudal part of brainstem and spinal cord (and consequently influences the sectioning plane of the brain most caudally). The brain used for the atlas was postfixed in the skull with 4% PFA (in 0.05 M PBS, pH 7.4) at 4 °C for 7 days, to best preserve the brain shape before removal and processing for cyto- and myelinated fiber architecture. The other brains were postfixed up to 24 h. Cryoprotection for freeze cutting was achieved by soaking the brains in 30% sucrose in 0.05 M phosphate buffer solution for 12 h. The brains were cut on a cryostat (LEICA CM 3050S, Leica Biosystems, Wetzlar, Germany) into four series of 40 µm thick frontal sections. The stain for acetylcholine-esterase was performed according to Hedreen (Hedreen et al. 1985). The NADPH-diaphorase stain followed the protocol of Vincent and Kimura (Vincent and Kimura 1992). The sections of the atlas brain were directly mounted on gelatin-coated slides and dried overnight. Alternating section series were stained on-slide either for cell bodies (Nissl) or for myelinated fibers (Gallyas 1979). Sections were imaged with a virtual slide microscope (VS120 S1, Olympus BX61VST, Olympus-Deutschland, Hamburg, Germany) at 10 × magnification using the proprietary software dotSlide® (Olympus).

### Atlas coordinate system

**Fig. 2:**
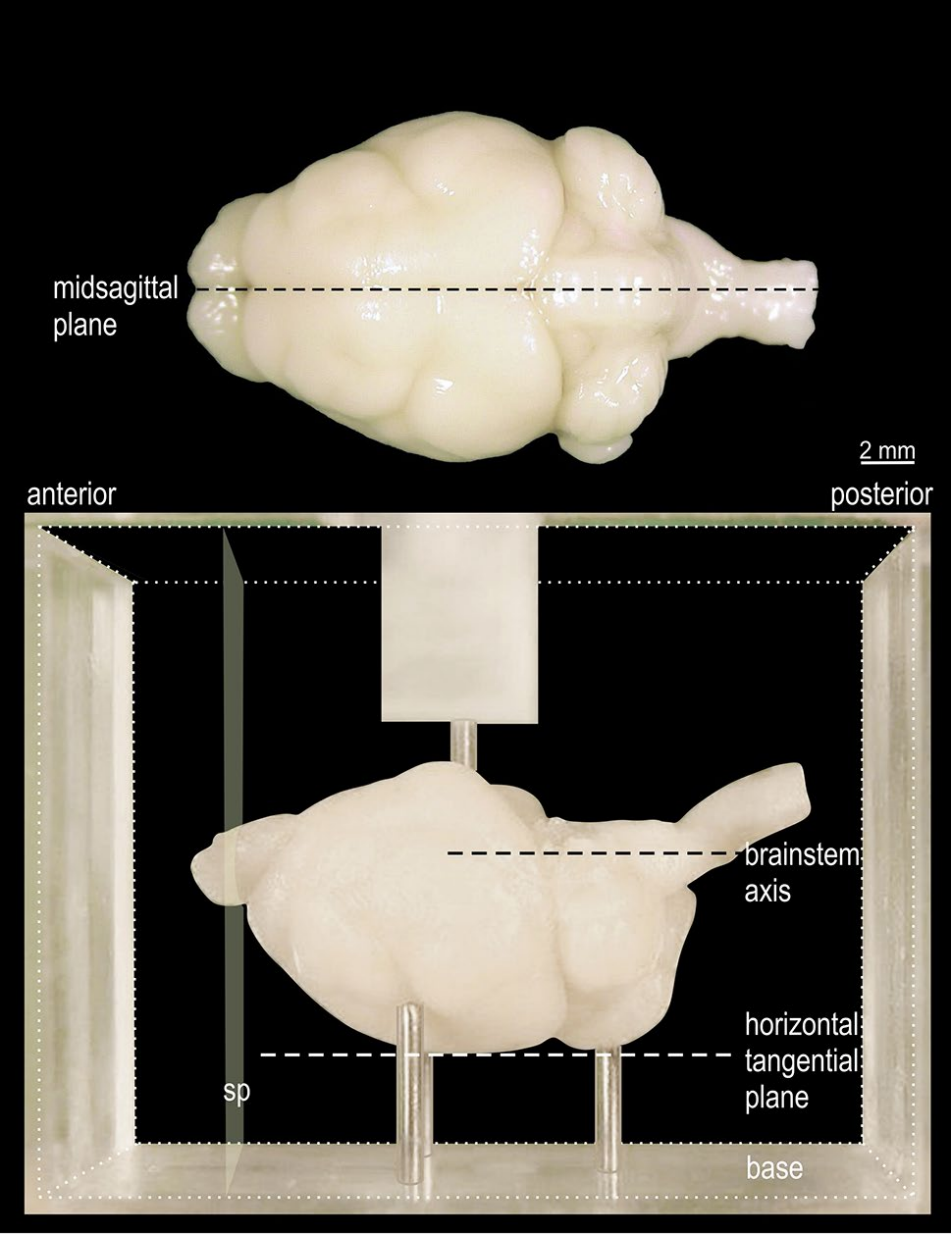
View of fixed *Phyllostomus discolor* brain, positioned for embedding. In the lower part of the figure the brain is shown in the acrylic glass box used for embedding (cubical volume indicated by fine dotted lines, front and back walls removed). The brain is positioned on 3 pins protruding from the base so that the plane defined by the most dorsal elevation of cerebrum and cerebellum (horizontal tangential plane) as well as the axis through the brainstem, are aligned parallel to the base. The anterior and posterior surfaces of the embedding block define the frontal sectioning plane (sp) perpendicular to the horizontal tangential plane and to the brainstem axis. A pin protruding from a bracket over the front and back walls of the box (only partly shown) prevents brain movement when the embedding medium is poured into the box. In the upper part of the figure the mid-sagittal plane is drawn in a top view of the brain

**Fig. 3:**
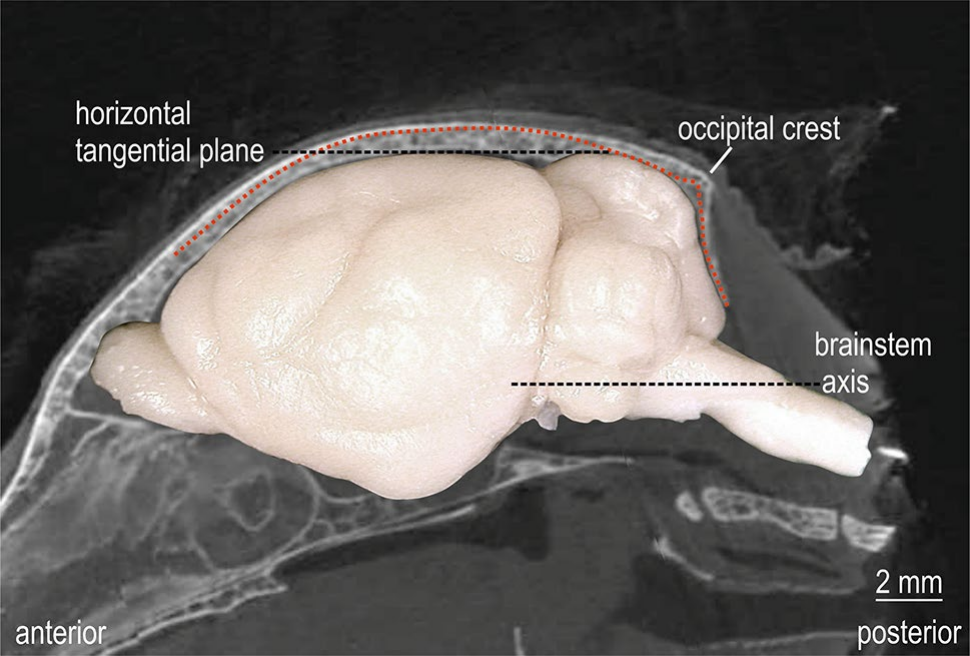
Atlas coordinate system and stereotaxic reference system. Montage of CT image of mid-parasagittal skull with the abrupt decline of occipital crest and the side-view image of the brain in standard orientation of the atlas. The red dotted line corresponds to the outer skull profile of the parasagittal CT image 1000 µm lateral to midline. The horizontal tangential plane through the most dorsal points of cerebrum and cerebellum and the brain stem axis is indicated by black dashed lines

The coordinate system of the *Phyllostomus discolor* brain atlas follows the conventional definition of anatomical sectioning planes in which frontal sections (‘sp’ in Fig. 2) are cut perpendicular to the brainstem axis (Fig. 2). In *Phyllostomus discolor*, the brainstem axis parallels the horizontal tangential plane passing through the most dorsal points of the cerebrum and the most dorsal point of the cerebellum (Fig. 2). This plane is chosen as the origin for the dorsoventral dimension of the coordinate system, with negative values in the ventral direction. The medio-lateral dimension is zeroed to the midsagittal plane (Fig. 2) with negative values on the right side, and positive values towards the left side of the head. There are two sets of coordinates for anterior–posterior position: the anterior–posterior position of the atlas brain plates is indicated relative to the rostral beginning of the neocortex (*y*_Start of Neocortex_ = 0; increasing values from anterior to posterior levels) and additionally, coordinates are given relative to the occipital crest (*y*_Occipital crest_ = 0; with decreasing values from caudal to rostral levels) as an external skull landmark (Fig. 3).

### Stereotaxic reference system

The coordinate system of the brain atlas was chosen to provide a viable baseline orientation of brain and head in a stereotaxic device. This orientation of the head enables a comfortable positioning of the animal and is suitable for electrophysiological experiments, tracer injections and optogenetic approaches. The alignment of the skull in the atlas brain coordinate system has been defined via CT. For this purpose, the CT scan was re-sliced in standard coordinates into 50 µm thick frontal slices with the open source program AMIDE: A Medical Imaging Data Examiner (amide.exe 1.0.4, ©Andreas Loening, https://amide.sourceforge.net/; GNU GPL).

Characteristic parasagittal outer skull profile lines of the CT are accessible during experiments and can be used to determine the baseline orientation of the skull. The occipital crest marks the abrupt decline of the occiput and is used as stereotaxic origin for the anterior–posterior coordinate (occipital crest, *y*_Occipital crest_ = 0). The parasagittal profile line and occipital crest coordinate, together with the symmetrical medio-lateral profile of the skull, define the baseline orientation of the skull in vivo to achieve a best fit of the brain in atlas coordinates. This profile-oriented stereotaxic procedure was described in detail in Schuller et al. (1986) and is recommended as standard adjustment procedure. It has been successfully used in *Phyllostomus discolor* in many studies involving forebrain and midbrain structures (Borina et al. 2008, 2011; Bartenstein et al. 2014; Fenzl and Schuller 2002, 2005; Firzlaff and Schuller 2007; Firzlaff et al. 2006, 2007; Greiter and Firzlaff 2017a, b; Genzel et al. 2015; Heinrich et al. 2011; Hörpel and Firzlaff 2019; Hoffmann et al. 2008a, b, 2010, 2013, 2015, 2016, 2019). These studies used a lab-internal series of Nissl stained sections that were relocatable to the present atlas series and fitted to the skull profile used here. The skull profile also matches well the contours of the cranium of *Phyllostomus discolor* presented by Kwiecinski (2006).

The stereotaxic standard position can be maintained throughout a series of experiments from the very beginning, by initially scanning the skull profile, then performing the experimental procedures in the stereotaxically oriented brain, and finally sectioning the brain in the same stereotaxic orientation as provided in the brain atlas. For this purpose the fixed brains are embedded in an acrylic glass box (Fig. 2, lower part) following a well-defined protocol (see Radtke-Schuller et al. 2016).

The protocol for positioning the brain for sectioning is straightforward. In short, the brain is positioned on the supporting needles of the embedding case so that the midsagittal plane of the brain is aligned parallel to the long side of the chamber, and that the virtual plane comprising the most dorsal points of the cerebrum and the cerebellum is parallel to the bottom plane of the embedding chamber. The latter adjustment provides a reproducible frontal sectioning orientation, whereas the former is important for the left–right symmetry of the sections. After stabilization of the brain in the desired position by a holding needle in a bracket from the top of the chamber, the embedding medium (a freshly prepared gelatin-albumin-glutaraldehyde or egg-yolk-glutaraldehyde mixture) is poured into the volume around the brain. Hardening of the block takes about 2–3 min. As the side walls, the holding bracket and the adjustable needles of the embedding chamber are detachable, the block can easily be removed from the box. After shock freezing in dry ice the block is directly mounted with its hind surface on the cutting platform of the cryostat. Due to the prior orientation of the brain in the embedding chamber the desired frontal sectioning plane is reached without further adjustment of the block in the cryostat. Further details of the embedding chamber and the procedure (developed for another bat species) can be found in Schuller et al. (1986).

Alternatively, it is also possible to section the brain in the standard atlas plane without embedding. In this case the brain is positioned upside down on a flat surface so that it is seated with the cerebellum and cerebrum on the base. Then part of the brain is cut off perpendicular to the base to create a surface for mounting the brain's portion of interest on the cryostat platform. By subsequent sectioning of the brain parallel to this cutting surface the resulting sections correspond best to the frontal plane of the atlas.

### Selection of atlas series and preparation of plates

The series for the new atlas consists of high-quality histological sections stained for cells (Nissl) and myelin (Gallyas) and matches the previous, successfully used, unpublished series.

To assess how representative the atlas series is within the pool of available *Phyllostomus dicolor* brain series (*N* = 7), the distance between rostral start and caudal end of the cortical hemispheres was evaluated for comparison. The average of this distance amounted to 11.093 mm with a standard deviation of 0.544 mm. The distance in the atlas brain is 11.086 mm, which signifies that the atlas brain is a valid representative of the average sized brain in this bat species. The comparison of the atlas brain series with the CT indicated a shrinkage of 8–9%, so that the virtual in vivo thickness of the sections (40 µm) amounts to 43.75 µm. This shrinkage is in the generally observed range for cryo-protected frozen-cut brains with PFA fixation.

There are 49 plates in the atlas. Nissl-stained and adjacent myelin-stained sections (Gallyas) at equidistant intervals of 350 µm (every 8th section) and every 7th CT section were taken to represent the 49 anterior–posterior (ap) levels. Distortions of the sections due to histological processing were compensated by cautiously adjusting the sections to optimize the congruency between outlines of histological sections and appropriate CT slices. Slight differences between the adjusted histology sections and the corresponding CT images do remain and can be judged by the overlay of structural delineations onto the CT as represented on the abbreviation subplates of the atlas. In the most caudal atlas plates (plate # > 45) the sections were less commensurate to the CT slices as the head/body angle of the atlas animal during perfusion fixation slightly deviated in orientation from that of the CT scan.

The contrast and brightness of the images of the sections were corrected with Photoshop (CS6, Adobe Systems, San Jose, CA, USA). The images were arranged in the atlas coordinate frame using CorelDraw graphics suite version 20 (2018) (Corel Corporation, Ottawa, ON, Canada). All outlines and delineations were drawn in CorelDraw on the base of the Nissl-stained section of each atlas plate. The structural boundaries seen in the corresponding myelin-stained section generally correlate well with these outlines.

**Fig. 4:**
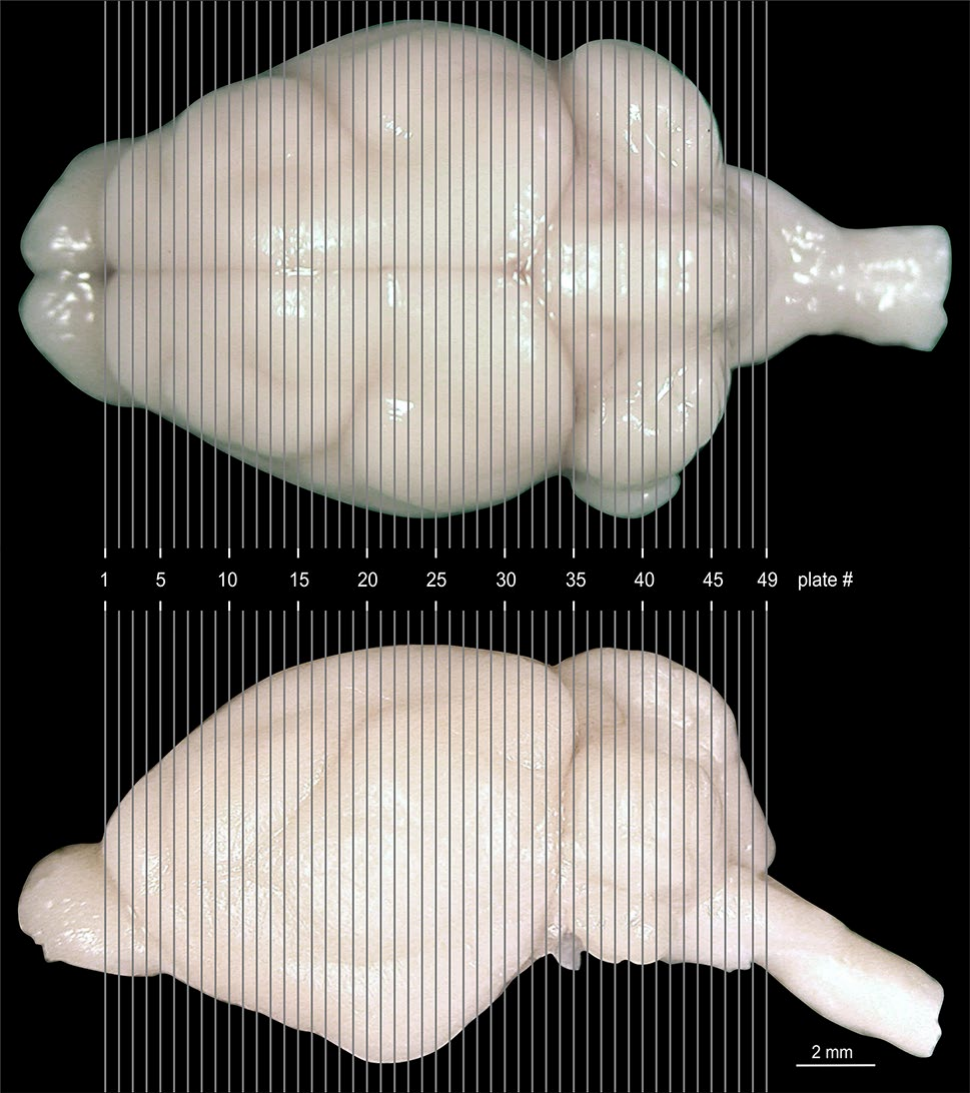
Anterior posterior location of the atlas plates on the *Phyllostomus discolor* brain indicated by gray gridlines. Top: view from above, bottom: side view. Distance between plates is 350 µm

An overview of the plate location in side and top view of the brain is presented in Fig. 4.

**Fig. 5:**
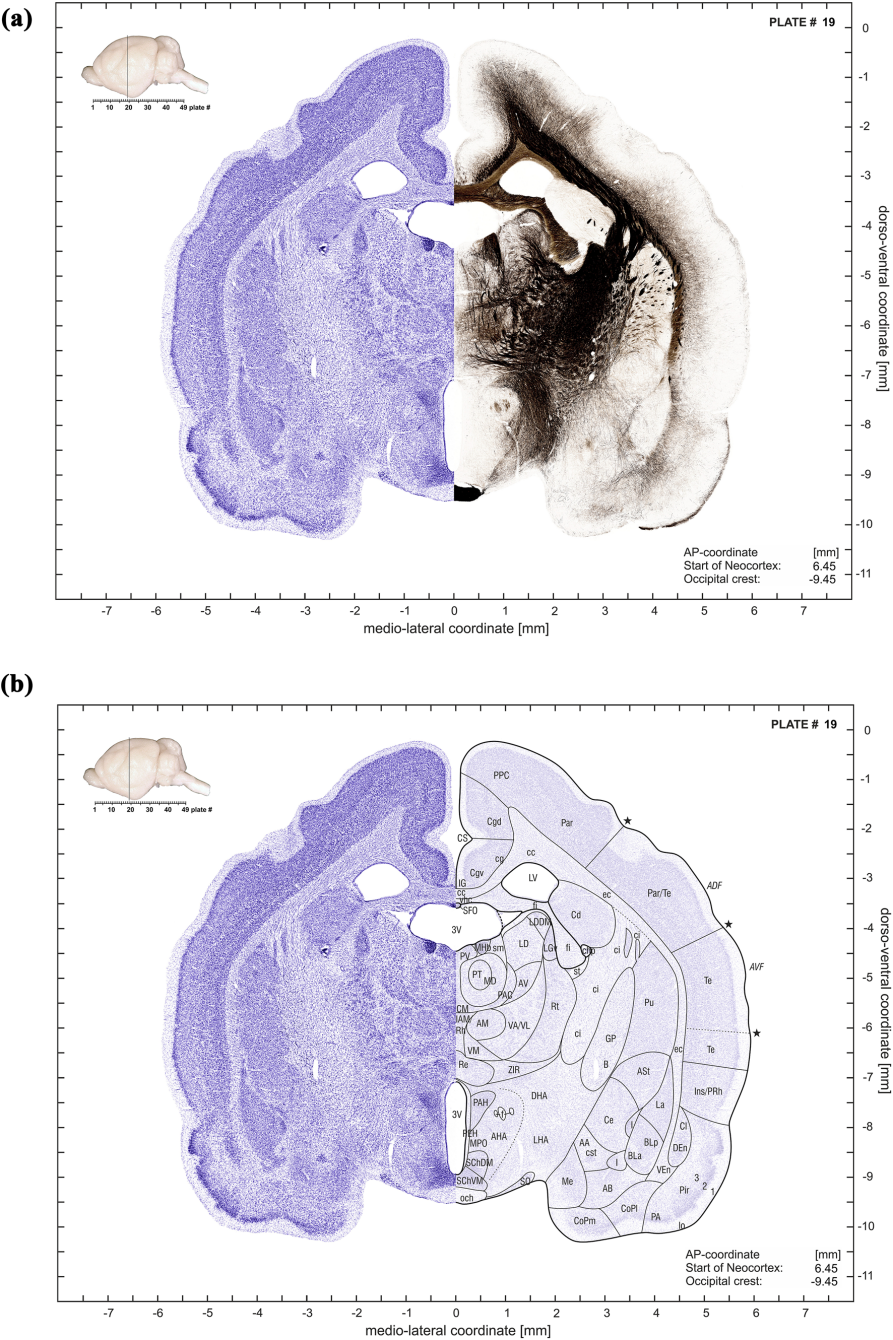

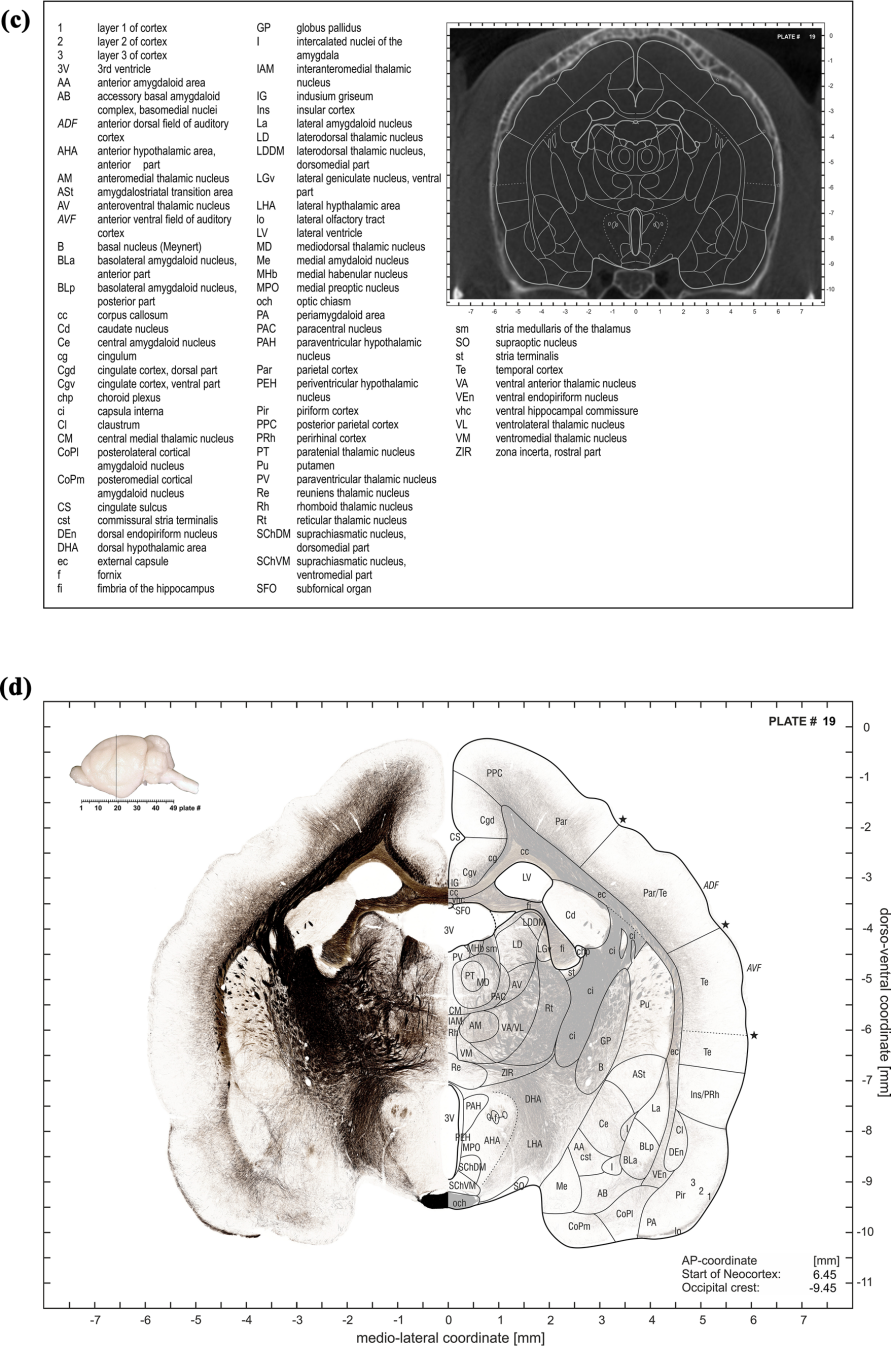
**a **First subplate of atlas plate 19. It consists of a montage of a Nissl-stained half-section with the mirrored adjacent myelin-stained half-section. The inset in the upper left corner indicates the anterior–posterior plate location in a lateral brain view which is also shown in numerical form relative to the most rostral “Start of Neocortex” as well as relative to the “Occipital crest” (in the lower right corner). **b** Second subplate of atlas plate 19. It combines the Nissl stained half section (left side) with delineations of the anatomical structures on the mirrored translucent (30%) Nissl section (right side). Inset and coordinate indications as in (**a**). **c** Third subplate of atlas plate 19. It consists of an abbreviation list and the CT slice with overlaid contours of the anatomical structures. **d** Fourth subpanel of atlas plate 19. It shows the myelin-stained half section (left side) with Nissl-derived delineations of the anatomical structures superimposed onto the mirrored translucent (30%) myelin-stained half section (right side). Inset and coordinate indications as in (**a**)

The plate at each ap-level consists of four subplates, as illustrated in the examples of Fig. 5a–d. The first subpanel depicts a montage of the Nissl stained half-section with the mirrored adjacent myelin-stained half-section (Fig. 5a). The second subpanel combines the Nissl stained half sections with delineations of the anatomical structures on the mirrored translucent (30%) Nissl section (Fig. 5b). The third subplate consists of an abbreviation list and the CT slice with the contours of the anatomical structures (Fig. 5c). The forth subplate shows the myelin-stained half sections with Nissl-derived delineations of the anatomical structures superimposed onto the mirrored translucent (30%) myelin-stained half section Fig. 5d).

The inset in the upper left corner of subpanels indicates the anterior–posterior plate location in a lateral brain view which is also shown in numerical form relative to the most rostral “Start of Neocortex”, as well as relative to the “Occipital crest” in the lower right corner of the 3 subplates 5A, B, D.

### Anatomical structures, nomenclature, and abbreviations

Anatomical structures were identified on the basis of cyto- and myeloarchitecture and by their relative locations. Additional brain series stained for chemo- and immunoarchitecture (calcium-binding proteins parvalbumin, calbindin and calretinin, and acetylcholine-esterase, acetylcholine-transferase, NADPH-diaphorase and zinc in various combinations) were consulted to support the structural identification. High-resolution images of one of these series with neighboring sections stained for NADPH-diaphorase, AChE and cells (Nissl) are presented as supplementary material (10.12751/g-node.8bbcxy). Examples for zinc stained sections are depicted in Hoffmann et al. (2008b).

**Fig. 6:**
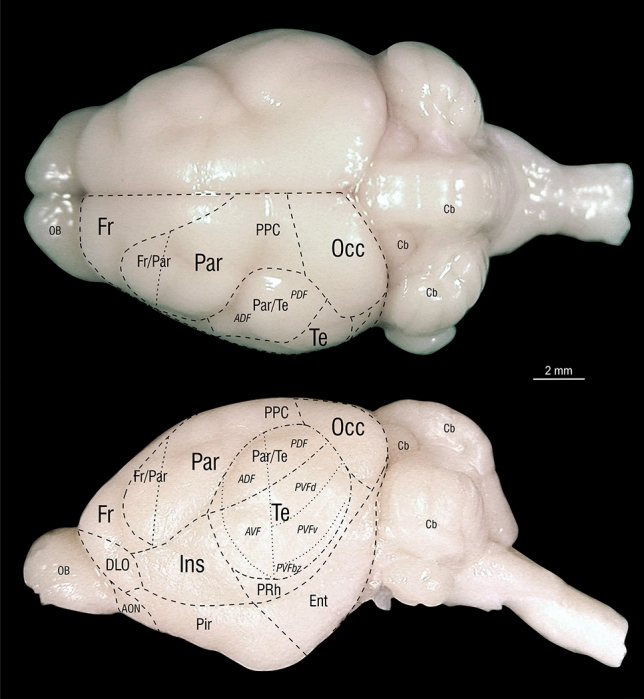
Delineations of structural and functional areas of the *Phyllostomus discolor* cortex in top and side views. Outlines are horizontal and lateral projections of the atlas brain delineations. Function-related terms are in italic letters; anatomical cortical structures are in standard letters. Abbreviations: *ADF* anterior dorsal auditory cortex field, AON anterior olfactory nuleus, *AVF* anterior ventral auditory cortex field, Cb cerebellum, DLO dorsolateral orbital cortex, Ent entorhinal cortex, Fr frontal cortex, Fr/Par frontal-parietal transition zone, Ins insular cortex, OB olfactory bulb, Occ occipital cortex, Par parietal cortex, Par/Te parietal-temporal transition zone, *PDF* posterior dorsal auditory cortex field, Pir piriform cortex, PPC posterior parietal cortex, PRh perirhinal cortex, *PVFbz* border zone of posterior ventral auditory cortex field, *PVFd* dorsal part of posterior ventral auditory cortex field, PVFv ventral part of posterior ventral auditory cortex field, Te temporal cortex

In general, since no unified neuroanatomical nomenclature exists to date (Swanson 2015), we have used the widely accepted Paxinos nomenclature and abbreviations for naming structures (as far as applicable) to ease comparison between species, including the rat (Paxinos and Watson 2007; Paxinos et al. 2009 and Zilles 1985 for cortex), the mouse (Franklin and Paxinos 2008; Watson and Paxinos 2010), the monkey (Paxinos et al. 2008), the gerbil (Radtke-Schuller et al. 2016) and the ferret (Radtke-Schuller et al. 2018). The already established terms for the AC fields of *Phyllostomus discolor* (Hoffmann et al. 2008b) were adopted and also mapped onto the atlas sections (borders marked by stars and field names are abbreviated in italic characters) and onto the brain surface (Fig. 6). Furthermore, we also adopted previous terms for auditory midbrain and brainstem nuclei for which bat specific terminology was already established. Abbreviations for nuclei and cortical regions are shown in uppercase characters, abbreviations for fiber tracts and fissures in lower case characters.

Delineations of functional and structural areas of the *Phyllostomus discolor* cortex in top and side views are depicted in Fig. 6. Outlines are horizontal and lateral projections of the atlas brain delineations in the plates.

An electronic version of the atlas plates, an index of abbreviations, an index of structures and the supplementary material is available at 10.12751/g-node.8bbcxy.

Additional publications on bat neuroanatomy (and those implicitly including neuroanatomical data on bats) taken into account for comparison are listed in the separate bibliography below ‘References’.
